# Estimation of parameters for plasma glucose regulation in type‐2 diabetics in presence of meal

**DOI:** 10.1049/iet-syb.2017.0036

**Published:** 2018-02-01

**Authors:** Prova Biswas, Ashoke Sutradhar, Pallab Datta

**Affiliations:** ^1^ Centre for Healthcare Science and Technology Indian Institute of Engineering Science and Technology Shibpur Howrah PIN 711 103 West Bengal India; ^2^ Department of Health and Family Welfare Institute of Pharmacy Jalpaiguri Swasthya Bhawan, Jalpaiguri PIN 735 101 West Bengal India; ^3^ Department of Electrical Engineering Indian Institute of Engineering Science and Technology Shibpur Howrah PIN 711 103 West Bengal India

**Keywords:** blood, diseases, biochemistry, parameter estimation, biological tissues, liver, Bayes methods, nonlinear filters, Kalman filters, drugs, drug delivery systems, medical signal processing, automated insulin delivery system, closed‐loop controller, hyperglycaemic patients, drug dose, root mean square error, cubature quadrature KF, Kalman filter, type II diabetes mellitus, process noise, Bayesian nonlinear filter, glucose‐insulin homoeostasis model, interstitial fluid, liver, portal vein, insulin mass, biological tissues, intestine, stomach, glucose mass, meal intake, type‐2 diabetics, plasma glucose regulation, parameter estimation

## Abstract

In this study, the authors propose a methodology for the estimation of glucose masses in stomach (both in solid and liquid forms), intestine, plasma and tissue; insulin masses in portal vein, liver, plasma and interstitial fluid using only plasma glucose measurement. The proposed methodology fuses glucose–insulin homoeostasis model (in the presence of meal intake) and plasma glucose measurement with a Bayesian non‐linear filter. Uncertainty of the model over individual variations has been incorporated by adding process noise to the homoeostasis model. The estimation is carried out over 24 h for the healthy people as well as a type II diabetes mellitus patients. In simulation, the estimator follows the truth accurately for both the cases. Moreover, the performances of two non‐linear filters, namely the unscented Kalman filter (KF) and cubature quadrature KF are compared in terms of root mean square error. The proposed methodology will be helpful in future to: (i) observe a patient's insulin–glucose profile, (ii) calculate drug dose for any hyperglycaemic patients and (iii) develop a closed‐loop controller for automated insulin delivery system.

## 1 Introduction

For a healthy human, through a series of physiological control action, plasma glucose level is maintained between 70 and 165 mg/dl throughout the day. When it decreases below certain value, blood supply to brain is restricted, which stimulates the release of glucagon, adrenalin and cortisol, thus increasing the plasma glucose concentration. In contrast, if plasma glucose concentration exceeds optimal range, insulin is released by action of hypothalamus on β cells and normoglycaemic condition is maintained by the stimulation of glucose transportation, utilisation and storage [1]. When the synthesis of insulin by β cell or sensitivity of cells to insulin is impaired, it leads to hyperglycaemic condition along with other complications such as uricosuria, ketoacidosis and negative nitrogen balance, collectively called as diabetes mellitus (DM) [2]. The associated chronic complications are diabetic neuropathy, diabetic nephropathy, diabetic retinopathy, diabetic foot ulcer, increased cardiovascular risks, diabetic coma *etc.* DM is primarily classified into two types. Type I diabetic patients are treated with insulin and type II diabetic patients are treated with oral hypoglycaemic agents in combination with insulin. Conventional blood sugar lowering drugs are major causes of death worldwide due to hypoglycaemia [2]. Moreover, it is reported that patients’ having blood glucose level below 50–60 mg/dl for 3 h or more in a day, suffer from arrhythmia, vasoconstriction, increased cytokine production and other associated complications [3–5]. So, it is necessary to maintain the glucose level within optimal range to prevent hyper and hypoglycaemia‐induced death.

A closed‐loop automated insulin delivery system, governed by control algorithm [6] [known as artificial pancreas (AP)] may become useful in maintaining blood glucose level within the limits prescribed [7]. An AP requires a control algorithm, which delivers only fixed amount of insulin. The success of controller mostly depends on accurate modelling which has been topic of continuous investigation for past few years. A mathematical model was developed by Srinivasan *et al.* [8], where the effect of fatty acid metabolism on plasma glucose–insulin concentration was illustrated. Later, Bergman proposed glucose–insulin homoeostasis model [9], where interrelation of plasma glucose, insulin and interstitial insulin was modelled. Furthermore, the model was modified by including insulin sensitivity and pancreatic responsiveness against intravenous glucose intake [10, 11]. Other researchers explored the impact of different hormones [12–14], different insulin delivery routes [15], glucose metabolism [16], oral [17] and intravenous glucose tolerance [18] on plasma glucose concentration. Papers on estimation and prediction of glucose and other related parameters using soft computing techniques such as artificial neural network, Fuzzy logic *etc.* [19–22] are also available in the literature.

Unfortunately, none of the methods considered the effect of glucose absorption from meal, which is obvious in real life. To address the shortcomings, a glucose hoemostasis model [23] has been developed for type I DM patients incorporating the rate of absorption of glucose from meal. Later on, another homoeostasis model [24] was proposed for type I DM patients, considering the rate of glucose appearance in plasma *via.* parenteral route. However, as the insulin production and release by the β cells are not included in these models, these could not be suitable to use for controlled delivery of insulin to type II DM patients. It is reported that basal insulin administration along with oral hypoglycaemic agents gives significant improvement in glycaemic control of type II DM patients [25]. Therefore, it became imperative to develop a model (incorporating meal) for any kind of hyperglycaemic persons including type II diabetes.

Cobelli *et al.* introduced a dynamic model [26] of glucose–insulin homoeostasis for normal as well as type II DM patients. The model considered necessary parameters such as oral glucose intake, its amount in stomach ($Q_{\text{sto}}(t)$), intestine ($Q_{\text{gut}}(t)$), plasma ($G_{p}(t)$) and tissue ($G_{t}(t)$); endogenous production of glucose by liver (EGP(*t*)), new insulin production in response to meal (*Y*(*t*)), insulin concentration in various body compartments such as portal vein ($I_{\text{po}}(t)$), hepatocytes ($I_{l}(t)$), hepatic vein ($I_{1}(t)$), plasma ($\left(I_{p}(t\right)$) and interstitial fluid (*X*(*t*)); insulin delay ($I_{d}(t)$) and their interconnecting relationship with plasma glucose level.

For treatment purpose, knowledge of the above‐mentioned parameters at any instant is required. The main constrain is that most of the blood glucose regulating parameters could not be measured. The challenge is to determine all the parameters without actually measuring them.

In this paper, a method has been proposed to estimate (know) the above‐mentioned parameters without actually measuring them with the sensor. It is true that the non‐linear filters are available in the literature, but their application to determine glucose regulating parameters is limited. More specifically, almost no work is available which describes a methodology to know glucose regulating parameters for type II diabetes patients in the presence of oral glucose intake.

Here, we propose a methodology which fuses the meal model (for both healthy persons and diabetic patients) with plasma glucose measurement with the help of two non‐linear filters, namely the unscented Kalman filter (UKF) and cubature quadrature Kalman filter (CQKF). The performance is compared in terms of root mean square error (RMSE), calculate out of 50 Monte Carlo (MC) runs. The filters estimate all the above‐mentioned physiological parameters at each instant of time in the presence of meal intake. Moreover, the plasma glucose level and other related physiological parameters vary considerably among individuals, depending on diet, duration of fasting, exercise, social and mental status, proper functioning of neuro‐endocrine system and body metabolic system [4]. To incorporate the variability, process noise has been added with the meal simulation model. Inaccuracy in glucose measurement sensor is modelled by sensor noise. Under such circumstances, it has been observed that the proposed methodology estimates the truth for both normal persons and diabetic patients.

## 2 Body compartments for glucose–insulin homoeostasis

**Fig. 1:**
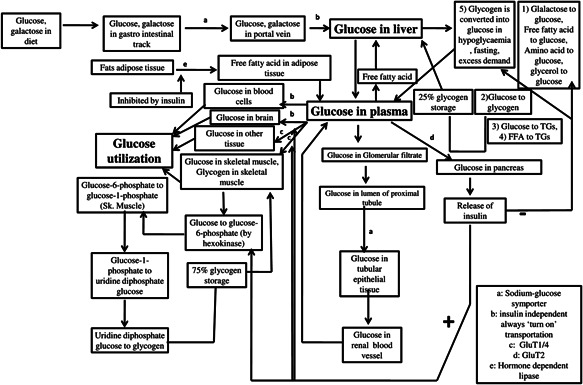
Fate of glucose in biological compartments

To understand the fate of glucose and insulin in human body at normal physiological condition, it is important to develop and validate a glucose–insulin homoeostasis model. The major sources of glucose in plasma are diet and glucose produced by the liver. The disappearance of glucose from plasma is associated with the utilisation by cells and storage in skeletal muscle and liver. Fig. 1 represents the fate of glucose in biological compartments. This section summarises the dynamic equations presented in [26, 27] with more physiological explanations.

### 2.1 Glucose subsystem

#### 2.1.1 Glucose absorption from intestine to extracellular fluid

After having a meal, solid glucose ($Q_{\text{sto}1}(t)$) is accumulated in the stomach and gradually converted into liquid glucose ($Q_{\text{sto}2}(t)$) to form a chyme. Later the chyme is transferred to the intestine through peristaltic movement three per minute. So, at any time, *t*, the total glucose present in the stomach $Q_{\text{sto}}(t)=Q_{\text{sto}1}(t)+Q_{\text{sto}2}(t)$. The rates of change of solid glucose and liquid glucose in stomach are given by

(1) $${\dot{Q}}_{\text{sto}1}(t)=-k_{\text{gri}}Q_{\text{sto}1}(t)+D\delta (t),$$

(2) $${\dot{Q}}_{\text{sto}2}(t)=-k_{\text{empt}}(Q_{\text{sto}})Q_{\text{sto}2}(t)+k_{\text{gri}}Q_{\text{sto}1}(t),$$

where $k_{\text{gri}}$ is the rate constant for the glucose grinding in stomach. δ(t) is a Dirac delta function, which is defined as infinite at *t* = 0 and 0 elsewhere with the area unity. It is worthy of mention here that the units of the all the constant values are listed in Table 1, hence not mentioned in the text individually. The constant, $k_{\text{empt}}(Q_{\text{sto}})$, is the gastric emptying rate. It is a non‐linear function of $Q_{\text{sto}}$, defined as [27]

$$\begin{array}{rl} k_{\text{empt}}(Q_{\text{sto}}) & =k_{\min}+\frac{k_{\text{max}}-k_{\min}}{2}\{\tanh[\alpha (Q_{\text{sto}}-bD)]\\ & \;-\tanh[\beta (Q_{\text{sto}}-cD)]+2\}, \end{array}$$

where α=(2.5/D(1−b)) and β=(2.5/Dc). $k_{\text{max}}$ is the maximum value of $k_{\text{empt}}(Q_{\text{sto}})$, in both the conditions, when *D* milligrams of glucose are present in stomach ($Q_{\text{sto}}=D$) or no glucose is present in stomach ($Q_{\text{sto}}=0$). $k_{\min}$ is the minimum value of $k_{\text{empt}}(Q_{\text{sto}})$. $\left(k_{\text{max}}-k_{\min}/2\right)$ is the value of $k_{\text{empt}}(Q_{\text{sto}})$, which exists in two situations, when, *b* and *c*% of meal (*D*) are present in stomach, *i.e.* $Q_{\text{sto}}(t)=c\times D$ and $Q_{\text{sto}}=b\times D$. Liquid glucose is transferred into the small intestine with the help of sodium glucose symporter, present in intestinal epithelium [5]. The rate of change of glucose in intestine is represented as

(3) $${\dot{Q}}_{\text{gut}}(t)=-k_{\text{abs}}Q_{\text{gut}}(t)+k_{\text{empt}}(Q_{\text{sto}})Q_{\text{sto}2}(t),$$

where $k_{\text{abs}}$ is the rate constant of glucose absorption from intestinal epithelium. It is considered that a fraction of the total absorbed glucose appears in plasma. So, the rate of glucose appearance in plasma, *Ra*(*t*) is calculated as per

$$Ra(t)=\frac{fk_{\text{abs}}Q_{\text{gut}}(t)}{\text{BW}}.$$

**Table 1:** Values of constants used in [26]

Processes	Parameters	Normal	Type II DM	Units
glucose kinetics	$V_{G}$	1.88	1.49	dl/kg
	$h/G_{b}$	75.177	181.4737	mg/dl
	$k_{1}$	0.065	.042	$m^{-1}$
	$k_{2}$	0.079	0.071	$m^{-1}$
insulin kinetics	$V_{I}$	0.05	0.04	l/kg
	$I_{b}$	25.556	59.875	pmol/l
	$m_{1}$	0.190	0.379	$m^{-1}$
	$m_{2}$	0.484	0.673	$m^{-1}$
	$m_{3}$	0.285	0.5685	$m^{-1}$
	$m_{4}$	0.194	0.269	$m^{-1}$
	$m_{5}$	0.0304	0.0526	$\frac{m\;\text{kg}}{\text{pmol}}$
	$m_{6}$	0.6471	0.8118	—
	$HE_{b}$	0.6	0.6	—
	$S_{b}$	1.549	4.027	pmol/kg/m
rate of appearance	$k_{\text{max}}$	0.0558	0.0465	$m^{-1}$
	$k_{\min}$	0.008	0.0076	$m^{-1}$
	$k_{\text{abs}}$	0.057	0.023	$m^{-1}$
	$k_{\text{Gri}}$	0.0558	0.0465	$m^{-1}$
	*f*	0.9	0.9	—
	*b*	0.82	0.68	—
	*c*	0.01	0.09	—
endogenous production	$k_{p1}$	2.7	3.09	$\frac{\text{mg}}{\text{kg}\;m}$
	$k_{p2}$	0.0021	0.0007	$m^{-1}$
	$k_{p3}$	0.009	0.005	$\frac{\text{mg}/\text{kg}\;m}{\text{pmol}/l}$
	$k_{p4}$	0.0618	0.0786	$\frac{\text{mg}/\text{kg}\;m}{\text{pmol}/\text{kg}}$
	$k_{i}$	0.0079	0.0066	$m^{-1}$
	$\text{EG}P_{b}$	1.8	2.1	mg/kg/m
utilisation	$F_{\text{cns}}$	1.0	1.0	mg/kg/m
	$V_{m0}$	2.5	4.65	mg/kg/m
	$V_{mx}$	0.047	0.034	$\frac{\text{mg}/\text{kg}/m}{\text{pmol}/l}$
	$K_{m0}$	225.59	466.21	mg/kg
	$K_{mx}$	0	0	mg/kg
	$P_{2u}$	0.0331	0.0840	$m^{-1}$
secretion	*K*	2.3	.99	$\frac{\text{pmol}/\text{kg}}{\text{mg}/\text{dl}}$
	*A*	0.05	0.013	$m^{-1}$
	*B*	0.11	0.05	$\frac{\text{pmol}/\text{kg}/m}{\text{mg}/\text{dl}}$
	γ	0.5	0.5	$m^{-1}$
renal elimination	$k_{e1}$	0.0005	0.0007	$m^{-1}$
	$k_{e1}$	339	269	mg/kg

Here, *f* is the percentage of absorbed glucose that reaches in plasma after *t* time and BW is the body weight.

#### 2.1.2 Glucose distribution in body compartments

Glucose transportation, use or storage in skeletal muscle and adipose tissue is dependent on insulin, expressed as $U_{\text{id}}(t)$, whereas its transportation into brain, red blood corpuscles, white blood cells, renal medulla and hepatocytes is independent of insulin, expressed as $U_{\text{ii}}(t)$ [4, 5]. The rate of change of $G_{p}(t)$ and $G_{t}(t)$ are expressed as

(4) $${\dot{G}}_{p}(t)=\text{EGP}(t)+Ra(t)-U_{\text{ii}}(t)-E(t)-k_{1}G_{p}(t)+k_{2}G_{t}(t),$$

(5) $${\dot{G}}_{t}(t)=-U_{\text{id}}(t)+k_{1}G_{p}(t)-k_{2}G_{t}(t).$$

*E*(*t*) is the elimination of glucose which is 0 in case of normal individuals. Glucose is eliminated in urine, if plasma glucose concentration exceeds renal threshold level, 339 mg/dl [26, 28]. $k_{1}$ and $k_{2}$ are the rate constants for the transfer of glucose from plasma to tissue and tissue to plasma, respectively. Plasma glucose concentration $G(t)=G_{p}(t)/V_{G}$, where $V_{G}$ is the volume of glucose distribution.

#### 2.1.3 Endogenous glucose production

Apart from diet, endogenous glucose production in liver is a major source of available glucose. About 20 mg/dl decrement from the basal value inhibits insulin release and glucose uptake in hypothalamus of brain that leads to release of several hormones, responsible for stimulation of endogenous glucose production [29–31]. EGP(t) is expressed as $\text{EGP}(t)=k_{p1}-k_{p2}G_{p}(t)-k_{p3}I_{d}(t)-k_{p4}I_{\text{po}}(t)$. Here, $k_{p1}$ is the extrapolated EGP(*t*) at zero glucose and insulin level in plasma. $k_{p2}$ is the liver glucose effectiveness, $k_{p3}$ is the parameter governing amplitude of insulin action on the liver $k_{p4}$ is the parameter, governing the amplitude of portal insulin action on liver. The rate of change of $I_{d}(t)$ could be expressed as

(6) $${\dot{I}}_{d}(t)=-k_{i}[I_{d}(t)-I_{1}(t)],$$

where $k_{i}$ is the rate parameter for delay between insulin signal and action. The change of $I_{1}(t)$ is written as

(7) $${\dot{I}}_{1}(t)=-k_{i}[I_{1}(t)-I(t)],$$

where *I*(*t*) is the plasma insulin concentration, calculated as $I(t)=(I_{p}/V_{I})$, $V_{I}$ is the volume of distribution of insulin.

#### 2.1.4 Utilisation of glucose

Total utilisation of glucose $U(t)=U_{\text{ii}}+U_{\text{id}}(t)$, where $U_{\text{id}}(t)=V_{m}(X(t))G(t)/K_{m}(X(t))+G_{t}(t))$. The first step of glucose utilisation is the conversion of glucose into glucose‐6‐phosphate by hexokinase, synthesised by the action of insulin. Glucose‐6‐phosphate is used for adenosine triphosphate generation in tissue and excess is converted into glycogen. Therefore, the utilisation of glucose in muscle is stimulated by insulin action. $V_{m}(X(t))$ is the reaction velocity of glucose conversion. It becomes faster in the presence of hexokinase, accelerated by insulin. $V_{m0}$ and $V_{mx}$ are the initial and maximum values of $V_{m}(X(t))$. The overall reaction velocity is: $V_{m}(X(t))=V_{m0}+V_{mx}X(t)$. The $K_{m}(X(t))$ is the Michaelis constant, dependent on the availability of the glucose [32]. The value of $K_{m}(X(t))$ at the initial point is $K_{m0}$. The reversible reaction stops after reaching to the equilibrium and the value of $k_{mx}$ collapses to 0 after a certain time and $K_{m}(X(t))=K_{m0}+K_{mx}X(t)$. *X*(*t*) is dependent on *I*(*t*) as well as basal insulin level, $I_{b}$, and expressed as

(8) $$\dot{X}(t)=-p_{2u}X(t)+p_{2u}[I(t)-I_{b}],$$

where $p_{2u}$ is the rate constant for insulin action on peripheral utilisation of glucose.

#### 2.1.5 Glucose elimination in urine

When plasma glucose reaches above the renal threshold $k_{e2}$, glucose starts getting eliminated in urine, which may be represented as

$$E(t)=\left\{\begin{array}{rl} & k_{e1}[G_{p}(t)-k_{e2}]\;\text{if}\;G_{p}(t)>k_{e2}\\ & 0\;\;\;\;\;\;\;\text{otherwise}, \end{array}\right.$$

$k_{e1}$ is the rate constant of glomerular filtration of glucose.

### 2.2 Insulin subsystem

The rate of change of $I_{p}(t)$ is

(9) $${\dot{I}}_{p}(t)=-(m_{2}+m_{4})I_{p}(t)+m_{1}I_{l}(t),$$

where $m_{1}$ and $m_{2}$ are the rate constants for insulin transfer from liver to plasma and plasma to liver, respectively. $m_{4}$ is the rate constant for peripheral degradation of insulin. The rate of insulin change in liver is written as

(10) $${\dot{I}}_{l}(t)=-(m_{1}+m_{3}(t))I_{l}(t)+m_{2}I_{p}(t)+S(t),$$

$m_{3}$ is the rate parameters for the degradation of insulin in liver cells. It depends on the hepatic clearance or extraction of insulin, HE(*t*) at that time: $m_{3}(t)=\text{HE}(t)m_{1}/(1-\text{HE}(t))$ . HE(*t*) is the ratio of irreversible hepatic efflux of insulin and total efflux of insulin from liver. At steady state, the insulin clearance *via* liver is 60%, that is, $HE_{\text{basal}}=0.6$ . Again, $S(t)=(m_{6}-\text{HE}(t)/m_{5})$, where $m_{5}$ is the rate constant for the transfer of insulin from pancreatic burst to portal vein [33]. $m_{6}$ is the rate constant for hepatic insulin clearance. *S*(*t*) is the total insulin secretion.

### 2.3 Insulin secretion

*S*(*t*) is directly proportional to $I_{\text{po}}(t)$, *i.e.* $S(t)=\gamma \cdot I_{\text{po}}(t)$. Here γ is the rate constant for insulin transfer between portal vein and liver. $I_{\text{po}}(t)$ follows the following differential equation:

(11) $${\dot{I}}_{\text{po}}(t)=-\gamma I_{\text{po}}(t)+S_{\text{po}}(t),$$

where $S_{\text{po}}(t)$ is the secretion of insulin above basal value and is expressed as

$$S_{\text{po}}(t)=\left\{\begin{array}{rl} & Y(t)+K\dot{G}(t)+S_{b}\;\text{if}\;\dot{G}(t)>0,\\ & Y(t)+S_{b}\;\;\;\;\;\text{otherwise}. \end{array}\right.$$

Here, *K* is the pancreatic responsiveness to rate of change of glucose. $S_{b}$ is the basal insulin secretion which follows $HE_{b}=-m_{5}S_{b}+m_{6}$. Now, replacing $S_{\text{po}}(t)$ in (11), we obtain

(12) $$\begin{array}{c} {\dot{I}}_{\text{po}}(t)=\left\{\begin{array}{rl} & -\gamma I_{\text{po}}(t)+Y(t)+K\dot{G}(t)+S_{b}\;\text{if}\;\dot{G}(t)>0,\\ & -\gamma I_{\text{po}}(t)+Y(t)+S_{b}\;\;\;\;\;\text{otherwise}. \end{array}\right. \end{array}$$

The model presented above is for the type II DM patients. Models of the type I DM are also available in the literature. The papers [23, 24] explicitly developed models for the type I DM patients. Moreover, many others models [8, 9, 12–16] could easily be modified for such patients.

As we know that type I DM patients cannot generate insulin in their body, the model used in the present paper can also be modified for type I DM patients. In that case we have to omit the equations describing insulin production and secretion. More explicitly, the model could be utilised successfully for type I DM patients if *Section 2.3* is removed.

## 3 Estimation of physiological parameters

### 3.1 Problem formulation

Let us assume the state vector, $x=[Q_{\text{sto}1}\;Q_{\text{sto}2}\;Q_{\text{gut}}\;G_{p}\;Y\;I_{\text{po}}\;I_{d}\;I_{1}\;I_{l}\;I_{p}\;G_{t}\;X]{\;}^{T}$. Equations (1)–(12) can be represented as $\dot{x}(t)=f(x(t))$, where *f*(.) is a non‐linear function. As we mentioned earlier, a process noise is added in the model to capture the uncertainty

(13) $$\dot{x}(t)=f(x(t))+\omega ,$$

where ω is the process noise; assumed as white and following a Gaussian distribution of 0 mean and *Q* covariance, *i.e.* ω∼N(0,Q). Plasma glucose is measured at a fixed interval of time. Measurement at any time step *k* is expressed as

(14) $$y_{k}=Hx_{k}+v_{k},$$

where H=[000100000000] is the measurement matrix and $v_{k}$ is the measurement noise assumed as Gaussian, *i.e.* $v_{k}∼N(0,\;R)$ and uncorrelated with ω. So, the problem reduces to estimate the state variable *x* from the noisy measurement (14). Here, we solve the estimation problem with two non‐linear filters, namely UKF and CQKF, which are the popular Bayesian filters in the literature.

### 3.2 Non‐linear Bayesian filters

The objective of the work is to estimate blood glucose regulating parameters continuously from inaccurate plasma glucose measurement. The problem is cast as a classical non‐linear estimation problem. Literatures exist [34, 35] to solve such biological problems with non‐linear filters, which use Bayes’ theorem and Chapman–Kolmogorov equation. These two equations combined together form a framework which is known as Bayesian framework of filtering.

For linear system and Gaussian noises those two equations could be solved analytically. However, for non‐linear systems those equations are intractable. Many approximate solutions are available in the literature which combined known as Bayesian non‐linear filter. Here, we used two of such filters, namely the UKF and CQKF.

#### 3.2.1 Unscented Kalman filter (UKF)

The UKF also known as sigma point KF approximates the posterior and prior probability density functions (pdfs) of states as Gaussian. The Gaussian pdf is characterised by few deterministically chosen quadrature points and weights associated with them. The main advantage of Gaussian filters is their computational efficiency. Mean value generated from points and weights are used to describe single point estimation. Now, we discuss the points and weights generation method.

In unscented transform method [36], the mean $\hat{x}$ and covariance $P_{x}$ of random vector ***x*** are evaluated with 2*n* + 1 sigma points (*n* is the dimension of the system) and its corresponding weights are as follows: assume 2*n* + 1 sigma points are scattered around mean in accordance to square root of the covariance matrix as

$$\begin{array}{rl} \xi_{0} & =\hat{x}\\ \xi_{i} & =\hat{x}+(\sqrt{(n+\kappa )P_{x}}){\;}_{i}\;i=1,\;2,\;\ldots ,\;n,\\ \xi_{i+n} & =\hat{x}-(\sqrt{(n+\kappa )P_{x}}){\;}_{i}\;i=1,\;2,\;\ldots ,\;n. \end{array}$$

In $(\sqrt{(n+\kappa )P_{x}}){\;}_{i}$, the subscript *i* represents the ith column or row of matrix $\left(\sqrt{(n+\kappa )P_{x}}\right)$. The weights of the sample points are evaluated as

$$\begin{array}{rl} W_{0} & =\kappa /(n+\kappa )\\ W_{i} & =1/2(n+\kappa )\;i=1,\;2,\;\ldots ,\;2n, \end{array}$$

where κ is the scaling parameter and its recommended value is κ=3−n for Gaussian distribution. Now, each sigma point is propagated through the non‐linear function

$$\xi_{i}=f(\xi_{i}).$$

Using the sigma points and its corresponding weights, the first two moments, namely mean $\hat{x}$ and covariance $P_{x}$ of vector ***x*** are computed as follows:

$$\begin{array}{rl} \hat{x} & =\sum_{i=0}^{2n}W_{i}\xi_{i},\\ P_{x} & =\sum_{i=0}^{2n}W_{i}(\xi_{i}-\hat{x})(\xi_{i}-\hat{x}){\;}^{T}. \end{array}$$

For detail algorithm, readers are requested to see [36].

#### 3.2.2 CQKF

The prior and posterior pdfs are approximated as Gaussian and realised with CQ points and weights [37]. The CQ points are generated from third‐order cubature and arbitrary‐order Gauss–Laguerre quadrature rule. The steps to generate CQ points and their corresponding weights are as follows:

- Find the cubature points $[u_{i}]{\;}_{\left(i=1,\;2,\;\ldots ,\;n\right)}$, located at the intersection of the unit hyper‐sphere and its axis.
- Solve the $n^{\prime }$‐order Chebyshev–Laguerre polynomial for α=(n/2−1) to obtain the quadrature points $\left(\chi_{i^{\prime }}\right)$
$$L_{n^{\prime }}^{\alpha }(\chi )=\chi^{n^{\prime }}-\frac{n{}^{\prime }}{1!}(n{}^{\prime }+\alpha )\chi^{n^{\prime }-1}+\frac{n{}^{\prime }(n{}^{\prime }-1)}{2!}(n{}^{\prime }+\alpha )(n{}^{\prime }+\alpha -1)\chi^{n^{\prime }-2}-\cdots =0.$$
- Find the CQ points as $\epsilon_{j}=\sqrt{2\chi_{i^{\prime }}}[u_{i}]$ and their associated weights as
$$W_{j}=\frac{1}{2n\Gamma (n/2)}(A_{i^{\prime }})=\frac{1}{2n\Gamma (n/2)}\frac{n^{\prime }!\Gamma (\alpha +n^{\prime }+1)}{\chi_{i^{\prime }}{\left[{\dot{L}}_{n^{\prime }}^{\alpha }(\chi_{i^{\prime }})\right]}^{2}}$$

for i=1,2,…,2n, $i^{\prime }=1,\;2,\;\ldots ,\;n{}^{\prime }$ and $j=1,\;2,\;\ldots ,\;2nn{}^{\prime }$.

Readers are requested to follow [37] for detail algorithm.

## 4 Simulation results

A software simulation of truth state variable and estimation is done for both the cases normal persons and type II diabetic patients. The selected case of study has some special importance. The model considered here is more realistic and almost all important parameters related to glucose–insulin homoeostasis are considered. Moreover, solid glucose meal intake as well as intake rate are taken into account. The case is studied for 24 h.

The values of the model parameters for both healthy and DM patients are taken as per Table 1 [26]. Truth states are realised by solving (1)–(12) with the initial values, summarised in Table 2. The BWs of normal individuals and diabetic patients are taken as 78 and 91 kg, respectively [26]. Both the cases, total 185 g of glucose are administered in three divided doses. About 37, 74 and 74 g glucose are served at breakfast, lunch and dinner, respectively, at the rate of 3.7 g/min. The simulation is started from 6 AM. The breakfast, lunch and dinner are served at 8 AM, 1 PM and 10 PM, respectively, shown with the bar plot in the secondary axis of the figures. The process noise covariance *Q* is assumed as $\text{diag}([1\;[0.1]{\;}_{1\times 11}])$. Measurement data are synthetically generated using MATLAB software. The measurement noise covariance is assumed as *R* = 16.

**Table 2:** Initialisation of truths and filters

Parameters	Normal	Type II DM	Units
stomach solid glucose ($Q_{\text{sto}1}$)	0	0	g
stomach liquid glucose ($Q_{\text{sto}2}$)	0	0	g
glucose in intestine ($Q_{\text{gut}}$)	0	0	mg
plasma glucose concentration ($G_{p}$)	75.18	181.47	mg/dl
new insulin production (*Y*)	0	0	pmol/l/m
insulin in portal vein ($I_{\text{po}}$)	6.04	6.38	pmol/kg
insulin delay ($I_{d})$	25.56	59.88	pmol/l
insulin in hepatic vein ($I_{1}$)	25.56	59.88	pmol/l
insulin in liver ($I_{l})$	4.57	5.95	pmol/kg
insulin in plasma ($I_{p}$)	25.56	59.88	pmol/l
tissue glucose ($G_{t}$)	106.16	144.46	mg/kg
interstitial insulin (*X*)	0	0	pmol/l

To estimate the states, the UKF has been implemented for both the healthy persons and DM patients. Later on the efficacy of the UKF is compared with the CQKF to estimate the states for normal individuals only. The filters are initialised from a Gaussian distribution with the mean values provided in Table 2, and initial error covariance $P_{0}=$ $\text{diag}([400^{2},\;0.1,\;0.1,\;0.1,\;200^{2},\;0.3,\;100,\;10,\;10,\;30,\;0.1,\;0.5])$. The sampling time of the state estimators is taken as 1 min. As the filters start from 6 AM after overnight fasting, initial glucose mass in stomach and intestine are taken as 0. The dimension of the system is 12, so there are 25 numbers of sigma points and weights are taken in case of UKF and 48 numbers of points are taken in case of CQKF.

**Fig. 2:**
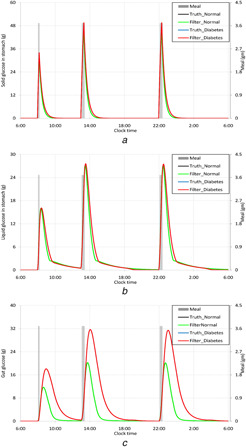
Estimation of glucose distributions in gastro‐intestinal track by the UKF ***(a)*** Solid glucose mass in stomach, ***(b)*** Liquid glucose mass in stomach, ***(c)*** Glucose in intestine

Figs. 2*a*–*c* represent the truth and estimated solid and liquid glucose masses in stomach and glucose mass in intestine for both, the healthy persons and DM patients. The figures show that the estimated values follow the truth. It is observed that after having a glucose meal there is no difference in rate of chyme formation in stomach in case of diabetic patients and normal people. It is justified, because the food degradation in stomach does not depend on the plasma glucose or insulin concentration. Once the chyme is formed in the stomach, it is absorbed in the plasma following its transfer into the duodenum. Chronic elevated blood glucose level causes the formation of glycated haemoglobin ($Hb_{A1c}$) and its deposition in capillaries of body tissues including gastric epithelial which further provokes the necrosis of corresponding tissues [2]. So, the autonomic reflex of gastro‐intestinal track is lost along with swelling in small intestine, which collectively called diabetic gastropathy [38]. As a consequence, the absorption of glucose from the intestine is delayed in chronic type II DM patients, that is, reflected in Fig 2c.

**Fig. 3:**
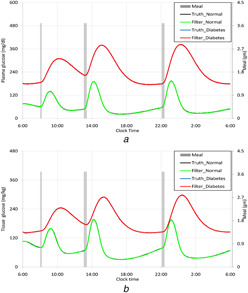
Estimation of glucose distributions in body by the UKF ***(a)*** Plasma glucose concentration ***(b)*** Tissue glucose mass

Figs. 3*a* and *b* represent the truth and estimated plasma and tissue glucose for healthy human beings as well as DM patients. It is observed that in both the cases the estimator tracks the truth. Insulin reduces plasma glucose by means of its transportation to liver, fat cells and striated muscle for utilisation as well as promoting its utilisation and inhibiting endogenous glucose production in liver. So, plasma insulin concentration is a rate limiting step for glucose transportation in muscle and fat cells and glucose utilisation in liver. In type II DM, the insulin responsiveness is impaired. Consequently, the plasma glucose concentration is increased markedly. The intervention of insulin is essential to use the glucose by various body tissues. When insulin sensitivity is impaired, the glucose uses by liver and skeletal muscle are interrupted. So, overall tissue glucose mass becomes high for chronic DM patients. This fact is reflected in figures.

**Fig. 4:**
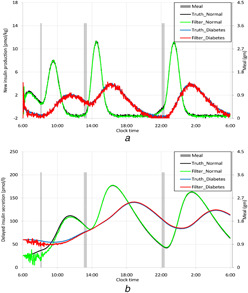
*Estimation insulin production and secretions by* β *cells, in two phases after oral glucose intake, by the UKF* ***(a)*** New insulin production after meal, ***(b)*** Delayed insulin secretion

**Fig. 5:**
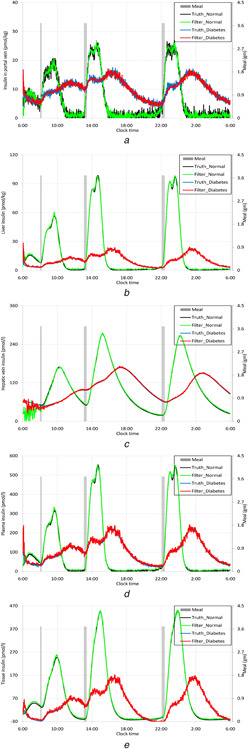
Estimation of insulin secretions by the UKF ***(a)*** Insulin mass in portal vein, ***(b)*** Insulin mass in liver, ***(c)*** Insulin concentration in hepatic vein, ***(d)*** Plasma insulin concentration, ***(e)*** Insulin concentration in interstitial fluid

Figs. 4*a* and *b* show the phases of new insulin production and delayed insulin secretion after meal intake. Insulin mass in portal vein and liver, insulin concentrations in hepatic vein, plasma and interstitial fluid are plotted in Figs. 5*a*–*e*. The estimator tracks the truth well. Insulin levels in portal vein, liver, hepatic vein and plasma for DM patients are maintained above normal basal range in compensation to insulin resistance. It is known that insulin is secreted in two phases in response to oral glucose administration. Glucose stimulates the secretion of glucagon like peptide‐1 and glucagon such as insulinotrophic peptide from gut, which stimulate the release of one fifth of the stored insulin from pancreas. The remaining stored insulin and newly synthesised insulin are secreted in the delayed phase in response to glucose absorption. The first phase of insulin secretion is impaired in case of the patients, persisting glucose level very high in plasma for long times. However, delayed insulin secretion remains unaffected in the disease condition. These figures support the biological facts.

**Fig. 6:**
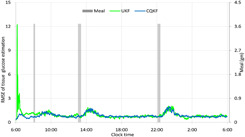
RMSE of estimation of glucose mass in tissue

**Fig. 7:**
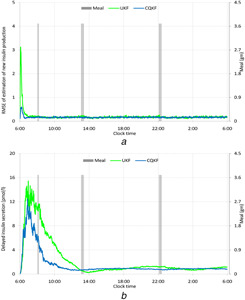
RMSE of estimation of insulin secretions in two phases after oral glucose intake ***(a)*** RMSE of estimation of new insulin production after meal, ***(b)*** RMSE of estimation of delayed insulin secretion

**Fig. 8:**
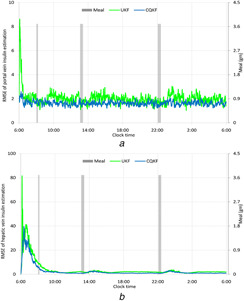
RMSE of estimation of insulin secretion in biological compartments ***(a)*** RMSE of estimation of insulin mass in portal vein, ***(b)*** RMSE of estimation of insulin concentration in hepatic vein

To compare the efficacy of two filters, we calculate the RMSE. The RMSE of the state, *x* at any instant *k* out of *M* MC runs is defined as $\text{RMS}E_{k}=\sqrt{(1/M)\sum_{j=1}^{M}{\left(x_{j,\;k}-{\hat{x}}_{j,\;k}\right)}^{2}}$. RMSEs of the tissue glucose mass, the insulin production and secretion by the β cells and the insulin levels in the portal vein and hepatic vein are plotted in Figs. 6–8, respectively. From RMSEs, it can be seen that both the filters converge to the truths successfully. However, the rate of convergence is little high for the CQKF. As both the filters show similar RMSEs for other states, so we did not include them as figures. The relative computational times of the two filters are given in Table 3. The computational time of the CQKF is almost double than that of the UKF.

**Table 3:** Compare of relative computational time of UKF and CQKF

Filter	Relative computational time
UKF	1
CQKF	1.97

## 5 Conclusion

In this paper, we proposed a methodology to estimate several biological parameters related to DM using only plasma glucose measurement. The proposed methodology combines the glucose–insulin homoeostasis model and plasma glucose measurement with the non‐linear Bayesian filters. It is assumed that only solid glucose is taken in oral route with a specific rate for a certain time. It is observed that both the filters converge to the truths successfully. However, the convergence rate is little high for the CQKF. On the contrary, the execution time for the CQKF is higher than the UKF. If the designer is ready to afford the higher computational time, the CQKF may be implemented. The UKF can also be implemented as only initial few minutes after instalment the estimation error is little high. Few minutes after installation, the UKF will converge to truth value and keep on following as long as the algorithm is not reset or the hardware is not removed from the patients’ body.

Using the proposed method, health care professionals could know about the variation of several biological parameters without actually measuring them. This would help them to take proper decision in managing the DM. Furthermore, the instantaneous values of the biological parameters will help the AP to calculate instantaneous dose of insulin, delivered through insulin pump.

The results presented in this paper are simulated with MATLAB software. However, in real‐time implementation, the programme must be embedded with hardware. The developed code can be written in hardware description language and it can be burned on a chip. The measurements will be stored in a buffer memory. The algorithm (implemented on hardware) will read the buffer and execute the programme. The hardware resources (which include memory, processors *etc.*) must be adequate to support the execution of the algorithm on hardware platform.
